# Emergence of Vancomycin-Intermediate *Staphylococcus aureus* and *S. sciuri*, Greece

**DOI:** 10.3201/eid0805.010387

**Published:** 2002-05

**Authors:** Athanassios Tsakris, Ekaterini Papadimitriou, John Douboyas, Fotini Stylianopoulou, Evangelos Manolis

**Affiliations:** * University of Thessaloniki, Thessaloniki, Greece; † University of Athens, Athens, Greece; ‡ AHEPA University Hospital, Thessaloniki, Greece

**To the Editor:** Staphylococcal isolates with reduced susceptibility to glycopeptides, such as vancomycin and teicoplanin, are a serious public health problem because staphylococci frequently show multidrug resistance, and glycopeptides are the only remaining effective drugs. Since the early reports of glycopeptide-resistant staphylococci, teicoplanin resistance has become more common than vancomycin resistance, particularly among coagulase-negative staphylococcal species (*1*–*3*). In cases of staphylococci with reduced susceptibility to vancomycin (vancomycin-intermediate staphylococci), an increasing number of strains showing heteroresistance are reported (strains that contain subpopulations of cells at frequencies >10^-6^ for which the vancomycin MICs are 8 µg/mL to 16 µg/mL); homogeneous resistance still appears to be rare (*2*,*4*–*7*). In northern Greece, resistance to teicoplanin has recently been documented in *S. haemolyticus* strains isolated from clinical infections (*8*). We report the first bloodstream infections in Greece associated with *S. aureus* and *S. sciuri* strains that have homogeneous intermediate-resistance to vancomycin (MIC = 8 µg/mL).

In our department, all clinically significant staphylococcal isolates are screened for reduced susceptibility to vancomycin and teicoplanin by an agar incorporation method (*9*), which has been routinely performed since January 1999. An inoculum of 10^4^ CFU/spot from a log-phase broth culture was spread on Mueller-Hinton agar plates containing appropriate antibiotic concentrations. The strains were incubated for a full 24 hours before the MICs were read. When a reduced susceptibility to vancomycin was observed (MIC 8 to 16 µg/mL), the test was repeated for confirmation of the result and the strains were also tested by National Committee for Clinical Laboratory Standards (NCCLS) broth microdilution (*9*) and E-test (AB Biodisk, Solna, Sweden) with BHI agar (Oxoid, Ltd., Basingstoke, Hampshire, UK) and an inoculum density adjusted to 0.5 McFarland value. *S. aureus* ATCC 29213, which had MICs for vancomycin of 1 µg/mL and for teicoplanin of 0.5 µg/mL, was used as a control for the estimation of the MICs. Two vancomycin-intermediate staphylococcal isolates (one *S. aureus* and one *S. sciuri*) were recovered in our hospital during December 2000 and April 2001, respectively. The organisms were identified with the Vitek system (bioMerieux Vitek, La Balme les Grottes, France). Slide-coagulase test and Staph ID 32 API system (API system, bioMerieux) confirmed identification. Susceptibility to 18 antimicrobial agents was evaluated with the Vitek system according to the recommendations of the manufacturer, and carriage of the *mecA* gene was confirmed with a polymerase chain reaction (PCR) that amplifies a 449-bp product.

The first strain (*S. aureus*) was recovered from a 52-year-old man who was hospitalized after a severe traffic accident. The patient had multiple injuries, including an external laryngeal trauma, pelvic ring disruption, and various fractures of the extremities. He underwent immediate tracheotomy, and a neurosurgical operation was performed to evacuate an extracerebral hematoma. Ceftazidime, clindamycin, ciprofloxacin, metronidazole, teicoplanin, and vancomycin were periodically administered as prophylaxis. An oxacillin-resistant *S. aureus* isolate was recovered from two blood cultures 4 weeks after the patient’s admission. The strain was also resistant to tobramycin, macrolides, tetracyclines, rifampicin, and fusidic acid, and had intermediate resistance to vancomycin (MIC 8 µg/mL) and teicoplanin (MIC 16 µ/mL) by all tested methods (agar dilution, broth microdilution, and E-test). The strain was susceptible to chloramphenicol, co-trimoxazole, fosfomycin, gentamicin, kanamycin, nitrofurantoin, and ofloxacin. The removal of an intravenous catheter and treatment with gentamicin and vancomycin eradicated the infection.

The second strain (*S. sciuri*) was recovered from a 35-year-old man who was an intravenous drug user. He was admitted with renal failure, electrolyte disturbances, and acute respiratory distress, which necessitated intubation and mechanical ventilation. The patient became febrile, and multiple courses of antibiotics (amikacin, cefepime, ciprofloxacin, metronidazole, and vancomycin, alone or in combinations) were administered before the *S. sciuri* strain was isolated. Seven weeks after his admission, an oxacillin-resistant *S. scuiri* strain that had cross-resistance to aminoglycosides, macrolides, quinolones, rifampicin, and tetracycline was found in subsequent blood cultures. The MIC of the strain for vancomycin was 8 μg/mL and for teicoplanin 16 µg/mL by the agar dilution method, and the result was confirmed by the E-test and the broth microdilution method. The strain was susceptible only to cotrimoxazole, fosfomycin, and nitrofurantoin. The patient improved clinically and was subsequently discharged on cotrimoxazole and vancomycin therapy.

In both cases, the MICs of vancomycin remained stable after repeated subcultures in a drug-free medium. PCR amplification showed that both staphylococcal strains carried the *mecA* gene. However, the *vanA*, *vanB,* and *vanC* genes were not amplified in any strain.

Vancomycin-intermediate staphylococci have been sporadically reported from clinical infections after prolonged exposure to vancomycin or preexisting infection with methicillin-resistant staphylococci (*5*,*7*). In our hospital, high rates of methicillin-resistant staphylococci are detected, and vancomycin has been the only treatment uniformly effective against staphylococcal infections. However, this is the first report of infection caused by vancomycin-intermediate *S. aureus* in Greece. In addition, *S. sciuri*, a species considered taxonomically the most primitive among staphylococci and found primarily in rodents and primitive mammals, has not been implicated previously in human infections caused by vancomycin-intermediate strains in our region or elsewhere.

Although various studies have described staphylococci with reduced susceptibility to vancomycin, the existence of isolates that have the homogeneous vancomycin-intermediate phenotype is rather limited (*4*–*7*). In this report, the vancomycin MIC for both staphylococcal isolates was repeatedly 8 µg/mL, and a confluent growth was observed after 24 hours on Mueller Hinton agar containing vancomycin at a concentration of 4 µg/mL. Discrete colonies were detected only in plates containing 6 μg/mL of vancomycin but not in plates containing 8 µ/mL of the drug even when a prolonged incubation of 48 hours and an inoculum of 10^6^ CFU/spot were used. The Vitek system recorded correctly both isolates as having intermediate level of resistance to glycopeptides; this result was particularly important given the wide use of this commercial system in many hospital laboratories. However, as was reported for previous glycopeptide-intermediate staphylococci (*2*,*5*), both isolates appeared to be susceptible to vancomycin when tested by the disk diffusion method, with zones of 15 mm and 16 mm for the *S. aureus* and the *S. sciuri* isolates, respectively.

Vancomycin-resistant staphylococci had not been detected in our hospital until December 2000. Therefore, the emergence of vancomycin-resistant staphylococci is a recent development, suggesting a potential for wider dissemination. Since the NCCLS agar dilution method we used is not sensitive for the detection of heterogeneous resistance phenotypes (*7*), screening for vancomycin-resistant subpopulations (mainly those with MIC for vancomycin of 4 µg/mL) in the vancomycin-susceptible isolates is important. Whether the mechanisms responsible for homogeneous intermediate resistance to vancomycin in our staphylococci are similar to those described in isolates from Japan and elsewhere still remains to be answered.
